# Effects of Elevated Temperature and Water Deficiency on Functional Traits of *Vitis vinifera* L. cv. Assyrtiko Leaves

**DOI:** 10.3390/plants14162463

**Published:** 2025-08-08

**Authors:** Foteini Kolyva, Ioannis-Dimosthenis S. Adamakis, Dimitrios Gkikas, Maria-Sonia Meletiou-Christou, Sophia Rhizopoulou

**Affiliations:** Section of Botany, Department of Biology, National and Kapodistrian University of Athens, 15784 Athens, Greece; iadamaki@biol.uoa.gr (I.-D.S.A.); jimgkikas@gmail.com (D.G.); mmeleti@biol.uoa.gr (M.-S.M.-C.)

**Keywords:** grapevine, morpho-anatomical traits, ecophysiological traits, phenolic compounds, sugars, starch, elevated temperature, water shortage

## Abstract

(1) Background: High temperatures and water scarcity, intensified by climate change, present major challenges to Mediterranean viticulture. In this study morphological, anatomical, and physiological responses of leaves at two developmental stages of *Vitis vinifera* L. cv. Assyrtiko were examined. (2) Methods: Young expanding and fully expanded leaves of two-year-old grapevines grown in pots in a controlled growth chamber, grown in soil in an open-air vineyard, and an adjacent greenhouse on Aegina Island (Greece), were used. The plants were subjected to water deficit (WD), elevated temperature (ET), and combined water deficit and elevated temperature (WD_ET) conditions for four months, and compared with control plants (C). (3) Results: Stress conditions caused contrasting effects on specific leaf area (SLA), which declined in young expanding leaves, except for a significant increase under elevated temperatures, while it increased in fully expanded leaves exposed to stress treatments. Concerning the anatomical traits, the thickness of spongy and palisade parenchyma did not change in young expanding leaves exposed to the three stress treatments, but increased in fully expanded leaves exposed to WD_ET. Metabolic traits (phenolic compounds, soluble sugars, and starch content) further highlighted these differences. (4) Conclusions: The findings reveal distinct stress responses between young expanding and fully expanded leaves of the grapevine Assyrtiko, underscoring the influence of water deficit and elevated temperatures on leaf developmental stage.

## 1. Introduction

The latest Intergovernmental Panel on Climate Change (IPCC) reports confirm that human-induced warming has increased global temperatures by 1.1 °C above pre-industrial levels, while projections under the high-emission pathway predict a rise from 3.3 °C to 5.7 °C, at the end of the 21st century [1]. The Mediterranean region is particularly vulnerable, facing a projected decline in precipitation (approximately 20%), intensifying prolonged drought periods and water scarcity [2,3,4]. Several studies suggest that viticulture in the Mediterranean region is highly vulnerable to climate change due to rising temperatures and decreasing water availability [5,6,7,8,9]. Also, viticulture is widely cited as a climate-sensitive agricultural system that has been used as an indicator of both historic and contemporary climate change, because of the long-term records [10,11]. Forecasts predict a future where regional climates will become increasingly mismatched with plants currently cultivated in wine-growing regions [12]. The maintenance of grapevine productivity, in the context of climatic change, will require the study of cultivars’ adaptation to climate change for the mitigation of these effects [10,13]. It is expected that with continued climate change that causes hotter and drier summers, certain varieties will start to fail, including yield declines and loss of cultivated lands [14]. It has been reported that vintners may need a transition to either grapevine cultivars better suited to warmer and drier climatic conditions or cultural practices that enhance grapevines’ resilience to challenging environmental conditions of climatic crisis through functional and structural changes [15]. A grapevine’s (*Vitis vinifera* L.) growth and development, physiological processes, yield, and quality are influenced by climate, and its vegetative cycle is highly controlled by temperature [16]. Although, warming and water shortage are the main abiotic stress factors, the effective functionality of grapevine cultivars exposed to water shortage and heat stress conditions has been affirmed [13,17,18,19]. However, the study of the leaf development of *Vitis vinifera* relevant to water shortage and increasing temperature is still limited. In this context, grapevine yield is curtailed by leaf area; also, the leaves provide functional substrate and substances for grapes’ development [20]. It should be noted that elevated temperatures coupled with water deficit affect grapevines’ physiology, morphology, and metabolism during the dry summer period in the Mediterranean region, which coincides with crucial growth phases of grapevines, e.g., the timing of flowering, leafing, ripe grapes, and harvesting [9,17].

The grapevine *V. vinifera* cv. Assyrtiko is a highly valued and well-known cultivar for viticulture in Greece, prized for its quality and adaptability. This white grape variety is indigenous to the volcanic terroir of Santorini Island in the Aegean Sea (Greece); also, it is widely planted on Cyclades’ Islands and cultivated in other regions of Greece, as well as in Australia and California [21,22,23]. It is noteworthy that own-rooted and phylloxera-free *V. vinifera* cv. Assyrtiko has been cultivated on the volcanic dry soil of Santorini for thousands of years [24,25,26]. *V. vinifera* cv. Assyrtiko is an early-ripening cultivar; i.e., grapes are harvested between July and August [27,28].

The young expanding leaves of *V. vinifera* cv. Assyrtiko are yellow-green with copper-colored patches, featuring a smooth upper surface and a pubescent abaxial side, while fully expanded leaves are wedge-shaped and symmetrical [29]. In fact, the grapevines’ leaf shape has historical importance [30]. Young expanding leaves are typically more sensitive to environmental stress due to their ongoing growth, extension, and development, while fully expanded leaves may have already undergone structural and functional adaptations to cope with abiotic stress [7,31]. As climate change intensifies, with rising temperatures and increasing water deficit in Mediterranean regions, differential responses between leaf developmental stages [32] could provide insights linked to the resilience of the *V. vinifera* cv. Assyrtiko. Plants respond to such abiotic stresses with anatomical changes, such as thicker leaves, compact mesophyll, reduced stomatal density, and altered mesophyll architecture, boosting water retention and cellular stability [22,33,34,35]. The leaves are sensitive to environmental changes and exhibit phenotypic plasticity as a response to abiotic stress [36,37,38]. Climate change affects the morphology and growth of grapevine leaves in specific ways that influence all developmental stages across species within the genus *Vitis* [39]. Although Assyrtiko is relatively adaptable to temperature fluctuations, prolonged exposure may affect leaf developmental stages [32,40]. For example, it has been observed that defoliation and water deficit decreased the weight and size, respectively, of Assyrtiko grapes [28,41], while harvest dates are arriving earlier during summer as grapes are maturing faster [42].

We recently described the structural and functional leaf traits of *V.s vinifera* cv. Assyrtiko plants under water deficit conditions, highlighting the dynamic role of CaO_x_ (druses and raphides) in relation to these traits [22], and the diffusion of CO_2_ in the leaves of the grapevines. These results raise new hypotheses for a better understanding of the processes governing grapevine leaf functionality and provide a framework for regulated development of an endemic, tolerant cultivar adapted to the future predicted climatic conditions in the Mediterranean region. In the present study, we examine how water deficit and an elevated temperature affect both the young expanding and fully expanded leaves of *V. vinifera* cv. Assyrtiko. This investigation is grounded in the premise that different leaf developmental stages may exhibit distinct responses to environmental stresses such as high temperatures and water scarcity. Specifically, in this study the influence of the above-mentioned abiotic stressors on leaf carbohydrate reserves and phenolic content—key factors linked to growth, adaptation, and defense mechanisms [43,44,45]—as well as on the specific leaf area and structural traits of the leaf blade [46,47]—was estimated. By comparing the responses of young expanding and fully expanded leaves to abiotic stress, our work emphasizes leaf adaptation in relation to leaf expansion that may affect different vintages of *V. vinifera* cv. Assyrtiko; also, it is expected to increase our understanding of the grapevine leaf developmental processes and plant response to predicted climate change in the Mediterranean region.

## 2. Results

### 2.1. Specific Leaf Area

**Figure 1:**
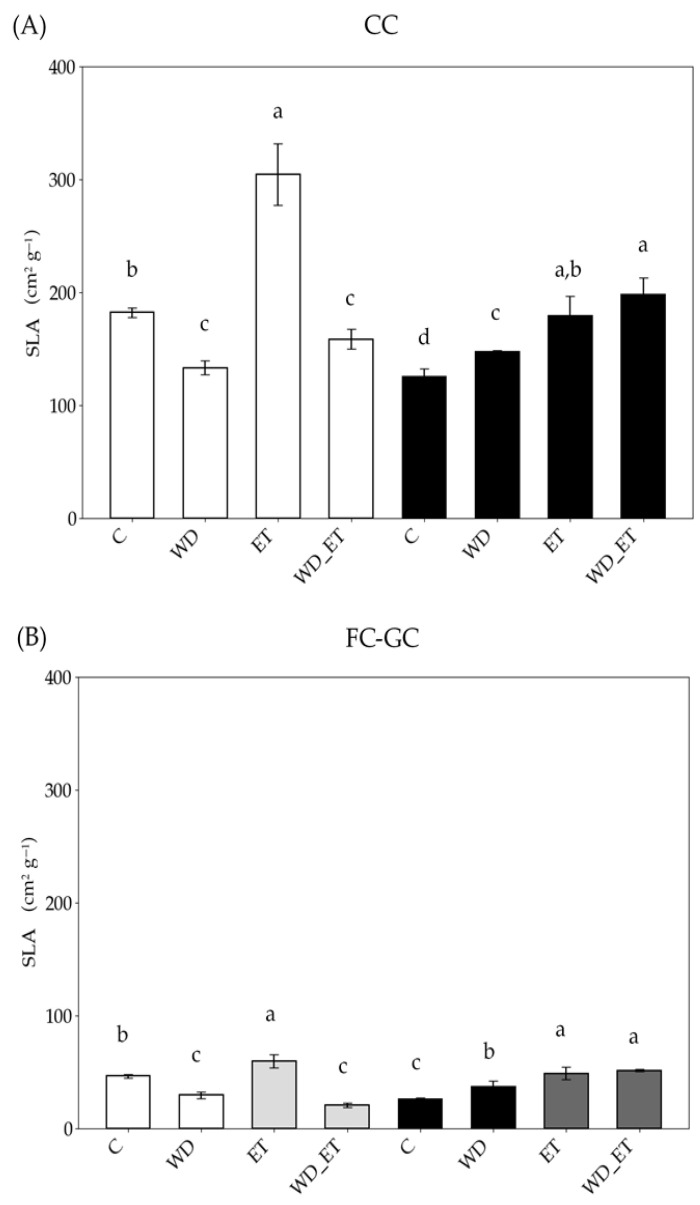
Specific leaf area (SLA) of *Vitis vinifera* cv. Assyrtiko control plants (C) and plants exposed to water deficit (WD), elevated temperature (ET), and a combination of water deficit and elevated temperature (WD_ET), and subjected to controlled conditions (CCs) (**A**) and field conditions and greenhouse conditions (FC-GCs) (**Β**). Young expanding leaves (white and light gray bars): in (**B**) white bars indicate FCs (field conditions) and light gray bars indicate GCs (greenhouse conditions). Fully expanded (black and dark gray bars): in (**B**) black bars indicate FCs and dark gray bars indicate GCs. The results are means of measurements from ten samples (n = 10) ± standard error (S.E.). Significant differences (*p* < 0.05) of mean values are marked using lowercase letters that are given separately for young expanding and fully expanded leaves.

The specific leaf area (SLA) of young expanding leaves decreased in plants grown under CCs (controlled conditions) and FC-GCs (field conditions and greenhouse conditions) and subjected to water deficit (WD) conditions, as well as to water deficit and elevated temperature (WD_ET) conditions (Figure 1); meanwhile, a substantial increase was observed in plants exposed to elevated temperature (ET) and subjected to both CCs (controlled conditions) and GCs (greenhouse conditions). In the case of fully expanded leaves, SLA increased in plants exposed to WD, ET, and WD_ET, and subjected to both CCs and FC-GCs (Figure 1), in comparison with the control conditions (CCs).

### 2.2. Anatomical Traits

#### 2.2.1. CCs

**Figure 2:**
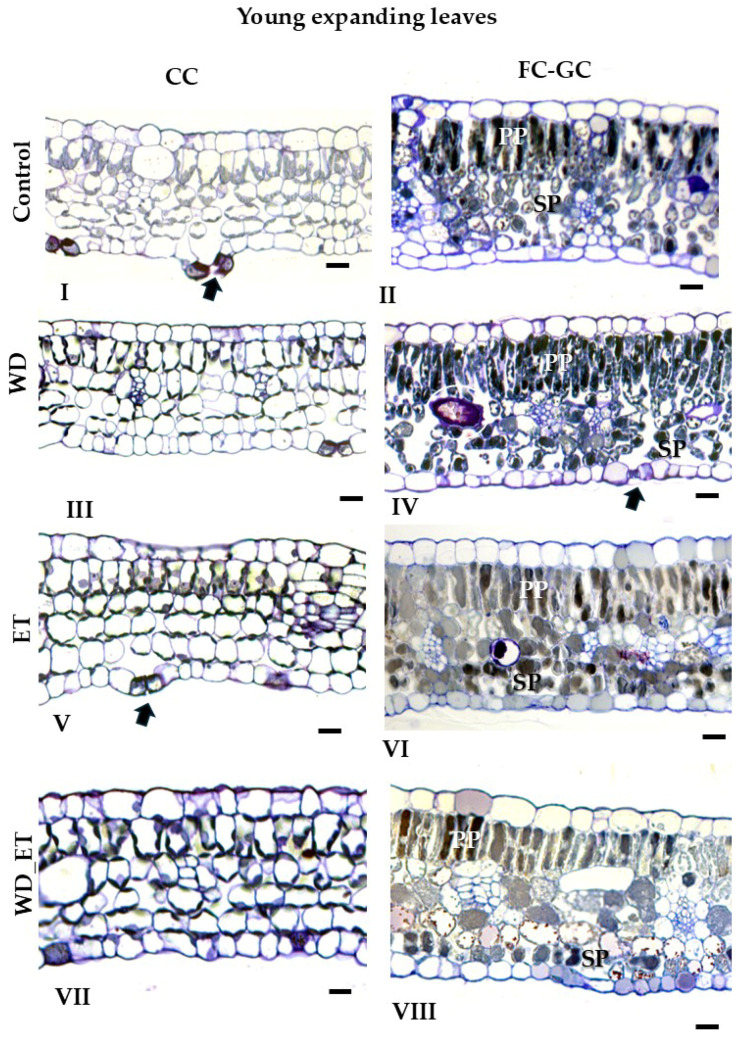
Toluidine blue-stained semi-thin cross-sections of young expanding leaves of *Vitis vinifera* cv. Assyrtiko plants subjected to GCs and FC-GCs; Roman numerals indicate leaf specimens of control plants (**I**,**II**), WD plants (**III**,**IV**), ΕT plants (**V**,**VI**), and WD_ΕT plants (**VII**,**VIII**). PP and SP indicate Palisade and Spongy parenchyma, respectively. Black arrows point to stomata. Scale bar: 20 μm.

**Figure 3:**
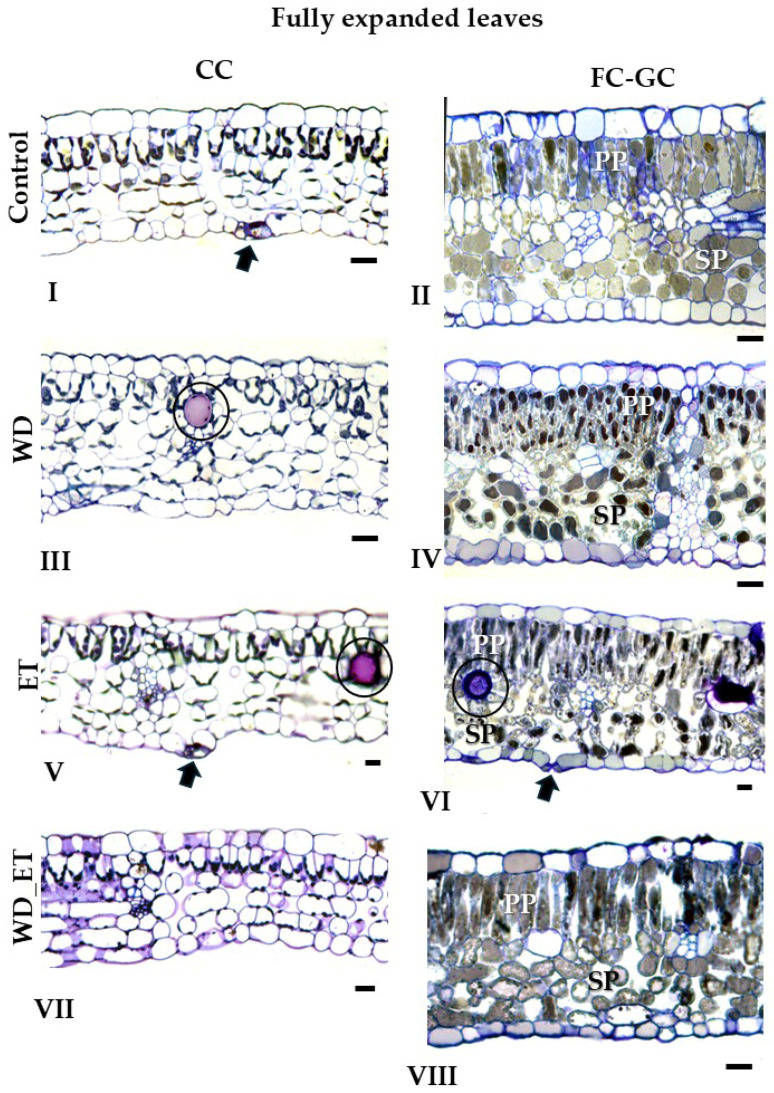
Toluidine blue-stained semi-thin cross sections of fully expanded leaves of *Vitis vinifera* cv. Assyrtiko plants subjected to GCs and FC-GCs; Roman numerals indicate leaf specimens of control plants (**I**,**II**), WD plants (**III**,**IV**), ET plants (**V**,**VI**), and WD_ET plants (**VII**,**VIII**). PP and SP indicate Palisade and Spongy parenchyma, respectively. Black arrows point to stomata. Black circles indicate idioblasts. Scale bar: 20 μm.

The thickness of young expanding leaves from treated plants (WD, ET, and WD_ET) subjected to CCs was quite similar (Figure 2, Table 1). The leaf thickness of fully expanded leaves significantly increased in ET plants, and a slight increase was observed in WD plants (Figure 2, Table 1). The thickness of the spongy mesophyll of young expanding leaves decreased in ET and WD_ET treatments (Figure 2, Table 1). In contrast, in fully expanded leaves, the thickness of the spongy parenchyma increased significantly in ET and WD plants, while it declined in WD_ET plants (Figure 2; Table 1). The thickness of the palisade parenchyma in young expanding leaves decreased in ET and WD_ET plants (Figure 2, Table 1), but increased in fully expanded leaves exposed to ET and WD treatments, while a slight decline was observed in WD_ET plants (Figure 3, Table 1). The intercellular space revealed significantly lower values in young expanding leaves of WD, ET, and WD_ET plants. However, in fully expanded leaves, the intercellular space increased in plants subjected to water deficit (WD) and elevated temperature (ET), while it decreased in WD_ET plants (Figure 3, Table 1).

#### 2.2.2. FC-GCs

The thickness of young expanding leaves from treated plants (WD, ET, and WD_ET) subjected to FC-GCs was quite similar (Figure 2, Table 1). The leaf thickness of fully expanded leaves increased in ET-treated plants grown under GCs, as well as in WD-treated plants grown in FCs, while a decrease was observed in WD_ET-treated plants grown in GCs (Figure 3, Table 2). The thickness of the spongy mesophyll of young expanding leaves was comparable among the induced stress treatments, thus quite similar values have been detected (Figure 3, Table 1). In contrast, in fully expanded leaves, the thickness of the spongy parenchyma increased significantly in ET- and WD-treated plants (Figure 3; Table 2). The thickness of the palisade parenchyma in both young expanding and fully expanded leaves was quite similar in treated plants (WD, ET, and WD_ET) subjected to FC-GCs (Figure 2 and Figure 3; Table 1 and Table 2). The intercellular space revealed significantly lower values in young expanding leaves of ET and WD_ET plants grown in CG (Figure 2; Table 1). Also, in fully expanded leaves, the intercellular space is reduced in plants subjected to WD_ET and ET (Figure 3; Table 2).

### 2.3. Metabolic Traits

#### 2.3.1. Phenolic Content

**Figure 4:**
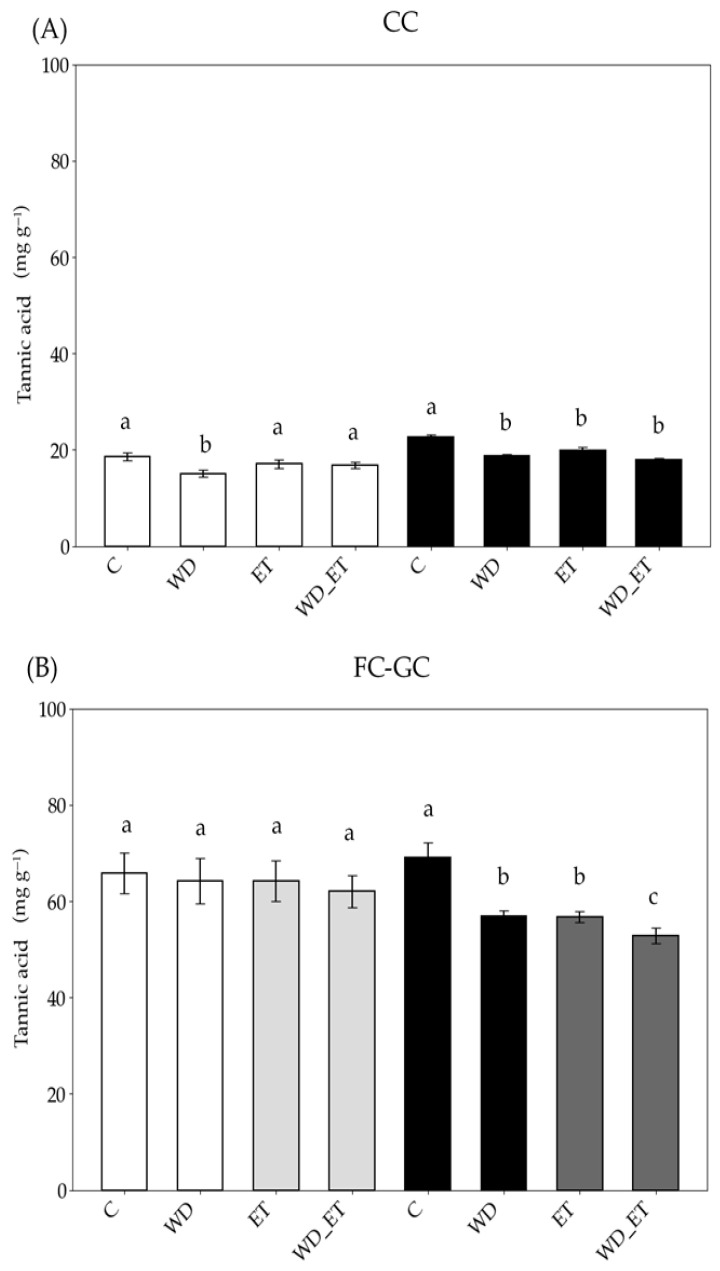
Concentrations of tannic acid in leaves of *Vitis vinifera* cv. Assyrtiko control plants (C) and plants exposed to water deficit (WD), elevated temperature (ET), and a combination of water deficit and elevated temperature (WD_ET), and subjected to CCs (controlled conditions) (**A**) and FC-GCs (field conditions and greenhouse conditions) (**Β**). Young expanding leaves (white and light gray bars): in (**B**) white bars indicate FCs (field conditions) and light gray bars indicate GCs (greenhouse conditions). Fully expanded (black and dark gray bars): in (**B**) black bars indicate FCs and dark gray bars indicate GCs. The results are means of five replicates (n = 5) ± standard error (S.E.). Significant differences (*p* < 0.05) of mean values are marked using lowercase letters that are given separately for young expanding and fully expanded leaves.

Under CCs, a reduction in the phenolic content was estimated in young expanding leaves exposed to WD, while in fully expanded leaves, significantly lower values (compared to the control) were estimated for the WD, ET, and WD_ET treatments (Figure 4). Under FC-GCs, the phenolic content was quite similar among young expanding leaves of the three treatments and comparable to that of the control plants (Figure 4A). However, a reduction in phenolic content was observed in fully expanded leaves subjected to WD, ET, and WD_ET conditions (Figure 4B). Additionally, the phenolic content of both young expanding and fully expanded leaves was approximately 3-fold higher in FC-GC-grown Assyrtiko plants in comparison with CC plants (Figure 4). Anatomical plates (Figure 2 and Figure 3) corroborate the quantified results, as phenolic compounds are visibly present in the cellular structures.

#### 2.3.2. Soluble Sugars

**Figure 5:**
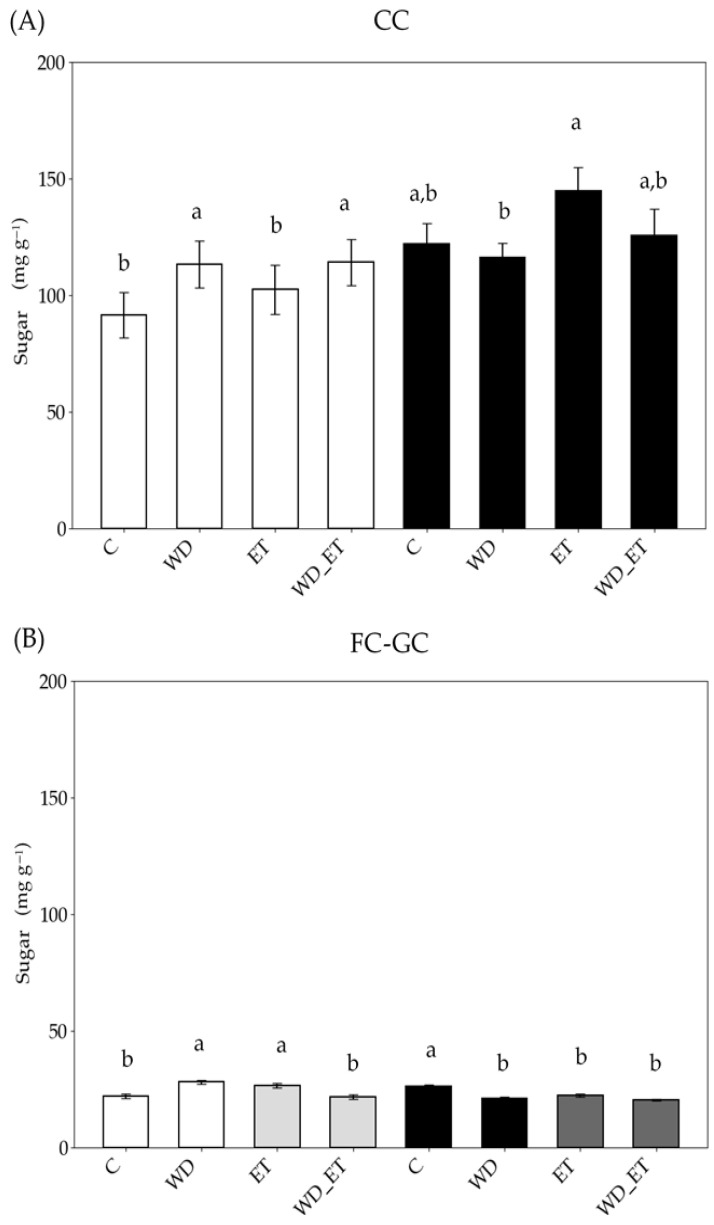
Leaf soluble sugar content of *Vitis vinifera* cv. Assyrtiko control plants (C) and plants exposed to water deficit (WD), elevated temperature (ET), and a combination of water deficit and elevated temperature (WD_ET), and subjected to CCs (controlled conditions) (**A**) and FC-GCs (field conditions and greenhouse conditions) (**Β**). Young expanding leaves (white and light gray bars): in (**B**) white bars indicate FC (field conditions) and light gray bars indicate GCs (greenhouse conditions). Fully expanded (black and dark gray bars): in (**B**) black bars indicate FCs and dark gray bars indicate GCs. The results are the means of five replicates (n = 5) ± standard error (S.E.). Significant differences (*p* < 0.05) of mean values are marked using lowercase letters that are given separately for young expanding and fully expanded leaves.

Under CCs, the soluble sugar content increased in the young expanding leaves of WD and WD_ET plants, while in fully expanded leaves an increase in soluble sugars was detected in ET plants (Figure 5). Under FC-GCs, the soluble sugar content increased in the young expanding leaves of WD and ET plants, while it declined in the fully expanded leaves of the three stress treatments, in comparison with the control plants (Figure 5). The sugar content of young expanding and fully expanded leaves was approximately 3-fold higher in Assyrtiko plants grown under CCs in comparison with FC-GC plants (Figure 5).

#### 2.3.3. Starch

**Figure 6:**
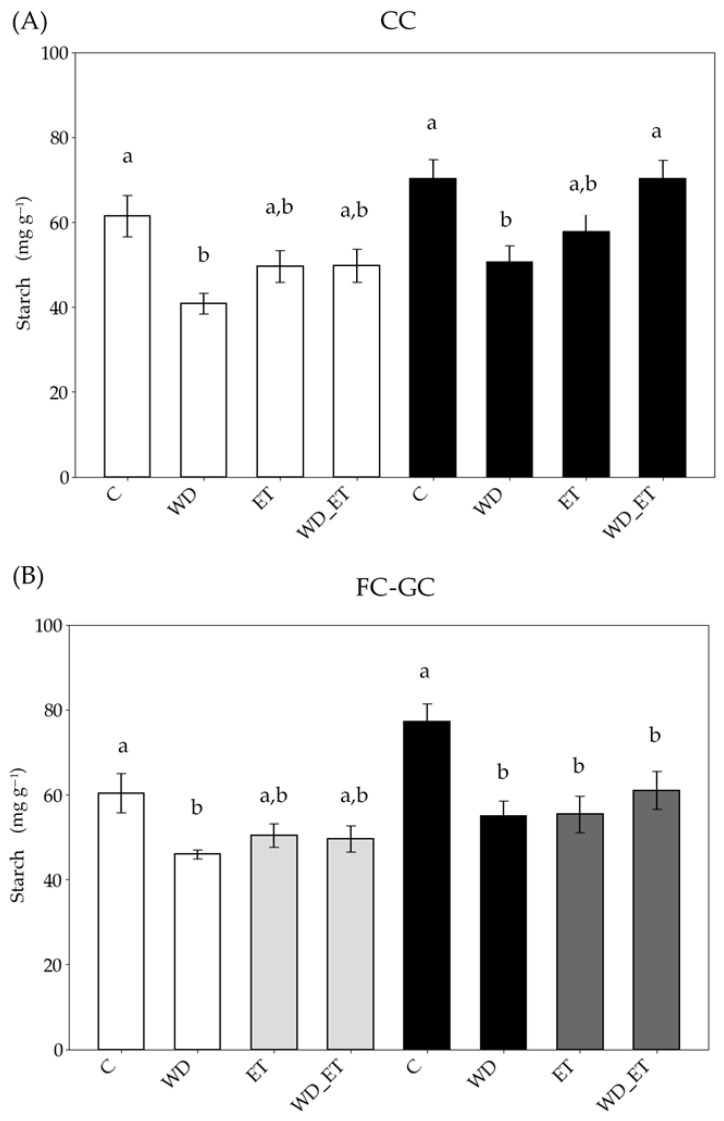
Leaf starch content of *Vitis vinifera* cv. Assyrtiko control plants (Cs) and plants exposed to water deficit (WD), elevated temperature (ET), and a combination of water deficit and elevated temperature (WD_ET) subjected to CCs (controlled conditions) (**A**) and FC-GCs (field conditions and greenhouse conditions) (**Β**). Young expanding leaves (white and light gray bars): in (**B**) white bars indicate FCs (field conditions) and light gray bars indicate GCs (greenhouse conditions). Fully expanded (black and dark gray bars): in (**B**) black bars indicate FCs and dark gray bars indicate GCs. The results are the means of five replicates (n = 5) ± standard error (S.E.). Significant differences (*p* < 0.05) of mean values are marked using lowercase letters that are given separately for young expanding and fully expanded leaves.

Under CCs, the starch content decreased in young expanding leaves of WD plants (Figure 6), and in fully expanded leaves of WD and ET plants. Under FC-GCs, the starch content decreased in young expanding leaves of WD plants, and in fully expanded leaves of the three treatments (significantly in WD and ET), in comparison with the control plants (Figure 6). Interestingly, the values of starch content of young expanding and fully expanded leaves from treated Assyrtiko plants grown in CC in comparison with plants grown in FC-GC were comparable (Figure 6).

### 2.4. Plasticity Index

The plasticity index (PI) of the intercellular area showed the highest values among the leaf traits (mean of 0.779), which suggests that despite the environmental changes (e.g., changes in temperature and irrigation conditions), young expanding and fully expanded leaves possessed a similar PI for intercellular spaces; this indicates that irrespective of variation in morphological PI, such as the thickness of palisade mesophyll and the low plasticity of the leaf thickness (mean of 0.223), the leaves tend to converge to similar photosynthetic capacity (Table 3). Thus, the low plasticity of the leaf thickness may be compensated by increased plasticity in other leaf traits. For example, the PI of photosynthates, such as sugars, was higher in young expanding than fully expanded leaves, and it was also two-fold higher (Table 3) in leaves of plants subjected to CCs (controlled conditions) in comparison with leaves developed under FC-GCs (field conditions and greenhouse conditions). In addition, the PI of starch was high. It is likely that the plasticity of SLA increased in both expanding and expanded leaf tissues exposed to field and greenhouse conditions, in comparison with that of the controlled conditions; the mean values in the case of young expanding leaves were 0.375 (CC) and 0.682 (FC-GC), and in the case of fully expanded leaves they were 0.417 (CC) and 0.593 (FC-GC).

### 2.5. Mixed-Effect Analysis

Mixed-effects modeling (Table 4) of individual leaf characteristics revealed highly significant baseline levels for the considered variables (intercept *p* < 0.001), with notable values for intercellular space (271.19) and SLA (101.35), which do indicate leaf developmental stage. A negative value of the leaf DS coefficient suggests that young expanding leaves exhibit lower values for the given variable compared to fully expanded leaves. Leaf developmental stage is significantly related to structural parameters, with young expanding leaves consistently showing lower values for intercellular space (−139.589), thickness of palisade parenchyma (−3.223), thickness of spongy parenchyma (−5.438), and leaf thickness (−9.226), in comparison to the fully expanded leaves. Among the ecophysiological parameters, starch content exhibited significant differences related to leaf developmental stage (−11.175, *p* < 0.001), while phenolic and sugar content remained stable. The high values of the group variance (treatment) suggest that different treatments caused large variation in certain traits; for example, intercellular space (3416.51), SLA (596.286), and starch content (62.532) showed high variability, which means that these traits were substantially affected by the stress treatments (Table 4).

The data presented in Table 5 demonstrate the effects of each treatment on leaf parameters. WD in CCs and FC-GCs significantly affected leaf thickness (*p* = 0.031), spongy parenchyma (*p* = 0.001), intercellular space (*p* = 0.024), and SLA (*p* = 0.003). According to our findings, physiological traits were unaffected by drought conditions, although a weak effect on sugar content was detected under WD conditions (*p* = 0.055). Additionally, the low *p* values related to spongy parenchyma (*p* = 0.001) and intercellular space (*p* = 0.001) suggest a substantial effect under ET conditions, while WD_ET conditions exert a significant impact on palisade parenchyma (*p* = 0.001), SLA (*p* = 0.001), and phenolic content (*p* = 0.024). Consequently, it is indicated that leaf structure is affected by abiotic factors, but since leaf DS is a fixed effect, it is assumed that young expanding and fully expanded leaves do respond differently, with structural traits being influenced more significantly than physiological traits.

### 2.6. Principal Component Analysis

**Figure 7:**
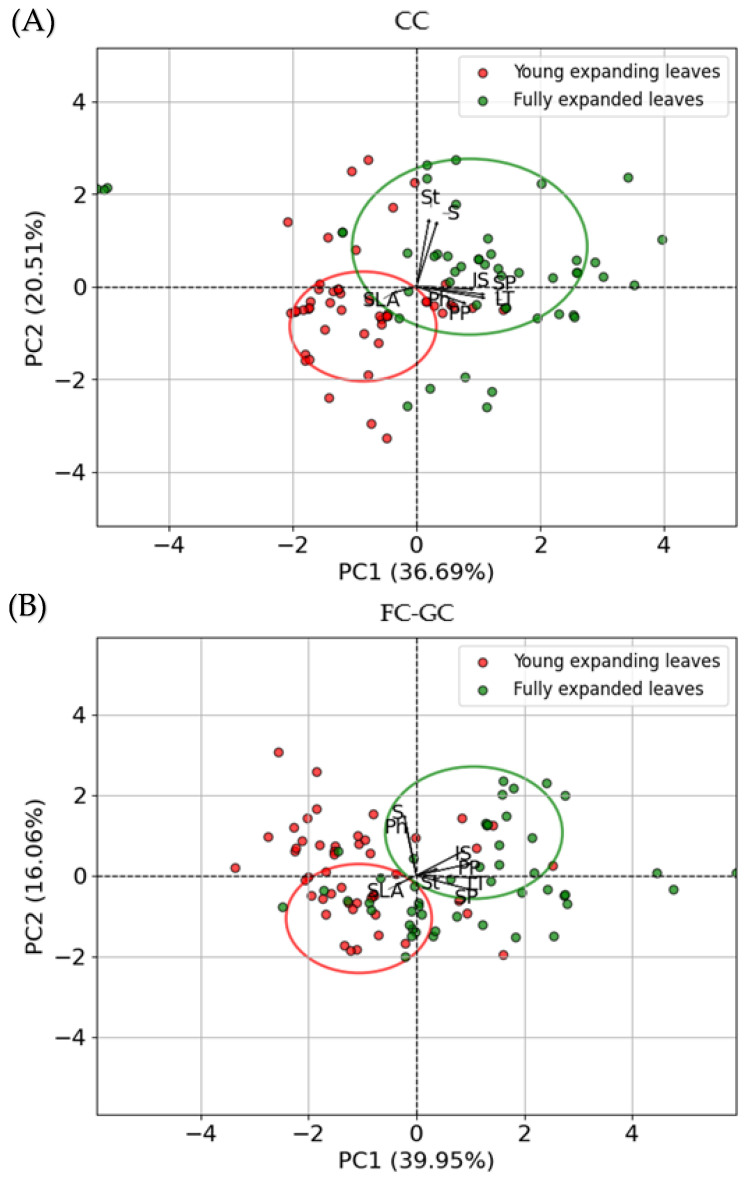
Visualization of PCA based on the considered traits of stress-treated plants of *Vitis vinifera* cv. Assyrtiko, revealing the grouping of specimens derived from young expanding (red) versus fully expanded (green) leaves for CCs (**A**) and FC-GCs (**B**). The abbreviated parameters in the diagram are: leaf thickness (LT), palisade parenchyma (PP), spongy parenchyma (SP), intercellular space (IS), specific leaf area (SLA), starch (St), phenols (Ph), and sugar (S).

The changes in parameters and leaves of grapevines *Vitis vinifera* cv. Assyrtiko were investigated by PCA (principal component analysis). PC1 alone explained 36.69% of the total variance and PC2 explained 20.51% of the total variance for CCs, while PC1 explained 39.95% and PC2 explained 16.06% for FC-GCs. The PCA results for CCs and FC-GCs indicate a differentiation between young expanding leaves and fully expanded leaves of *Vitis vinifera* cv. Assyrtiko in treated plants (Figure 7). PCA plots showed clear separation between the two leaf stages, with fully expanded leaves clustering in the right region of the plot, while young expanding leaves were positioned in the left region of the plot.

The PCA axes account for a substantial percentage of the variation, indicating that the chosen variables (e.g., phenols, sugars, starch, etc.) contribute significantly to the differentiation between leaf stages. The considered anatomical and ecophysiological traits indicate a considerable grouping of the leaf specimens collected from treated plants grown under regulated (Figure 7A), ambient, and greenhouse conditions (Figure 7B). Under both CCs and FC-GCs, PC1 is primarily associated with anatomical variables, which were found to be grouped together indicating a strong positive correlation, contributing similarly to the principal components. PC2 was found to be associated with physiological variables, i.e., sugars and starch content under CCs, while sugars and phenolic content in FC-GCs along with SLA. Notably, SLA points in the opposite direction than all the anatomical traits suggesting negative correlation with those variables (Figure 7).

## 3. Discussion

The anatomical response of grapevine leaves to water deficit and elevated temperature demonstrated plasticity, particularly in young expanding leaves, under water deficit (WD), elevated temperature (ET), and the combined treatment (WD_ET), a significant reduction in the thickness of intercellular spaces, palisade, and spongy mesophyll was observed in young expanding leaves. It is expected that young, developing leaves are sensitive to abiotic stresses, because they are still undergoing expansion (unpublished data) and, thus, are more susceptible to reduced water availability [48]. The morphological response is a common adaptation of plants subjected to drought stress, where a modified leaf structure may reduce water loss [49]. The observed decrease in intercellular space also supports the aspect that young expanding leaves may reduce respiratory water loss and optimize CO_2_ uptake under stress, providing an effective mechanism for coping with elevated temperature and water deficit conditions [50]. Moreover, these structural adjustments may help the plant to conserve energy and resources during stressful periods, ensuring better growth and survival in the face of changing environmental conditions. However, under an elevated temperature (ET), leaf thickness, spongy parenchyma, and intercellular space increased in fully expanded leaves, indicating a different adaptive strategy. The increase in thickness under heat stress may be an adaptive advantage of retaining sufficient water content, as thicker leaves might exhibit a higher capacity to store water and resist excessive evaporation under elevated ambient temperatures [35,51,52]. This anatomical adaptation has been frequently observed in plants exposed to heat stress and is linked to reducing water loss while maintaining turgor pressure [33,48,49,50,51,52,53,54,55].

These findings align with previous studies [47,56,57], where drought and heat stress induced anatomical changes in plants, such as thicker epidermal layers and denser cellular arrangements, aiming at a reduction in water loss [58]. The mixed-effects model analysis showed that leaf developmental stage played a significant role in anatomical responses, with young expanding leaves of Assyrtiko consistently displaying smaller intercellular spaces and reduced mesophyll width, which may represent a limitation to carbon assimilation and photosynthesis. The PCA further validated these results, revealing clear separation between the anatomical responses of young expanding and fully expanded leaves (Figure 7), suggesting that the leaves’ developmental stage significantly influences their ecophysiological and anatomical characteristics. The considered parameters reflect the cultivar’s efficiency to optimize water retention during periods of water stress [59,60].

The specific leaf area (SLA) of young expanding and fully expanded leaves exhibited different responses to drought and heat treatments, indicating the role of leaf developmental stage; in fact, SLA decreased in young expanding leaves of WD- and WD_ET-treated plants exposed to controlled (CC) and field and greenhouse (FC-GC) conditions. This decline suggests a restriction in leaf expansion, potentially as a strategy of maintaining efficient water status [61]. A smaller SLA is often associated with thicker leaves, which may improve water use efficiency and enhance biomass allocation, as well as mechanical stability under stress conditions [62]. However, SLA increased in young expanding leaves of plants subjected to ET treatments, indicating an alternative adaptive strategy, prioritizing leaf expansion, and enhancing light capture in response to elevated temperatures [63,64]. The SLA of fully expanded leaves increased in plants subjected to ET, WD, and WD_ET conditions, under both CCs and FC-GCs; this increase suggests that fully expanded leaves undergo anatomic and metabolic modifications in order to maintain their functionality under the above-mentioned abiotic factors.

The response of grapevine leaves to water deficit and elevated temperature has been reflected in significant changes in soluble sugar and starch content. Soluble sugars support osmoregulation and the maintenance of turgor [65]; also, they protect cellular structures from dehydration and oxidative damage, ensuring that cellular processes continue under stress conditions [66,67]. Soluble sugars, which act as osmotic regulators helping the plant regulate its water potential and prevent dehydration [66,67,68,69], were found to increase in young expanding leaves in most treatments, with the exception of the elevated temperature treatment under controlled and greenhouse conditions; this increase is consistent with the role of soluble sugars in osmotic adjustment. The mixed-effects model analysis confirmed that soluble sugars were highly responsive to treatment effects, with significant variation observed particularly in young expanding leaves. This finding suggests that young expanding leaves are involved in metabolic adjustments to maintain turgor and osmotic balance under abiotic stress [70]. Soluble sugar content was also increased in fully expanded leaves grown under CCs and subjected to elevated temperature, indicating a potential role in osmotic regulation. In contrast, the soluble sugar content of fully expanded leaves decreased in plants grown under FC-GCs, and subjected to ET, WD, and WD_ET conditions. Thus, a differential response between the two different developmental leaf stages has been detected, where younger leaves accumulate soluble sugars under drought and heat stress in contrast to fully expanded leaves.

The decrease in leaf starch content detected in this study aligns with previous studies reporting a reduction in starch reserves during drought conditions, possibly due to the mobilization of starch into soluble sugars to facilitate leaf osmotic adjustment [71,72,73,74,75]. Starch is typically broken down into simple sugars to maintain osmotic balance and ensure cellular function under stress conditions [76]. It has been argued that the simultaneous reduction in starch and increase in soluble sugars may sustain turgor and cellular function under drought and heat stress [77].

Interestingly, the phenolic content, which plays a critical role in protecting plants from oxidative damage under stress, was found to be approximately 3-fold higher in both young expanding and fully expanded leaves of plants grown in field/greenhouse conditions, in comparison with that of plant leaves grown in regulated conditions. In controlled, field, and greenhouse conditions, the phenolic content in young expanding leaves declined only in the case of plants subjected to WD treatment. However, the pronounced reduction observed in fully expanded leaves under combined stress conditions (WD_ET) suggests that such conditions (i.e., drought and elevated temperature) may impair the synthesis of phenolic compounds, therefore reducing the plant’s ability to counter oxidative damage and maintain its leaf longevity [78,79,80].

According to the mixed-effect modeling, both leaf developmental stage and treatment significantly influenced anatomical traits, i.e., intercellular space, palisade and spongy parenchyma, and leaf thickness; actually, the young expanding leaves possessed lower values than those of fully expanded leaves. Also, leaf developmental stage affected the starch content. Soluble sugars and phenolic content were found to be linked to declining though more stable values in fully expanded leaves compared to young expanding leaves; it seems likely that fully expanded leaves that exhibit more complete metabolic pathways—as is also indicated by SLA—may tolerate environmental stressors better than the young expanding leaves. In the case that leaf growth is forced by elevated temperatures, carbohydrates could be remobilized [81,82,83].

Young expanding leaves actively adjust their physiological traits through flexible metabolic processes to counter immediate abiotic stress, while fully expanded leaves rely on pre-established mechanisms that allow them to endure prolonged stress with minimal metabolic shifts. This divergence—reflected in the SLA values—underscores the importance of considering both anatomical and metabolic traits, when evaluating grapevine adaptation under combined water deficit and elevated temperatures, recognizing the critical role of leaf developmental stage in plant response to stresses.

## 4. Materials and Methods

### 4.1. Plant Material and Experimental Conditions

#### 4.1.1. Controlled Conditions

**Figure 8:**
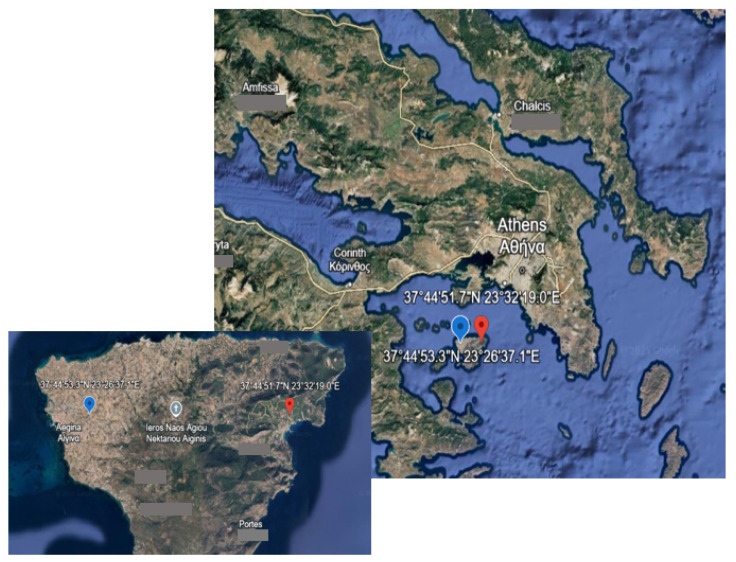
Maps of the experimental site on the island of Aegina (Aίγινα) located south of the capital city of Athens (Aθήνα) and southeast of the city of Corinth (Κόρινθος) in Greece (upper-right map), where the red dot indicates the location of the experimental vineyard (37°44′51.7″ N, 23°32′19.0″ E), and the blue dot indicates the meteorological station on Aegina Island (37°44′53.3″ N, 23°26′37.1″ E), which is presented magnified in the insert—left map.

**Figure 9:**
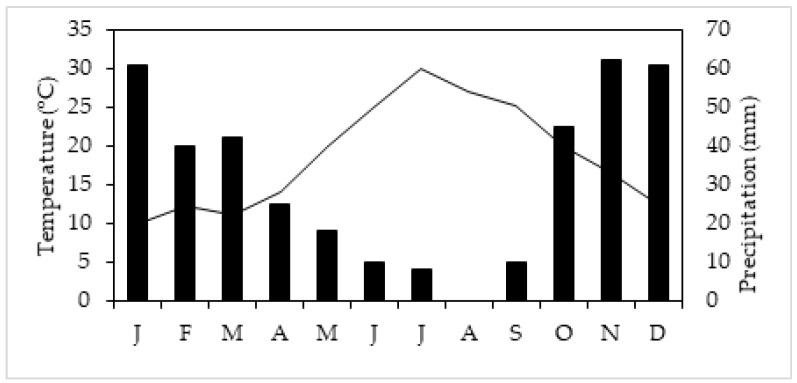
Ombrothermic diagram (Precipitation scale: 2 × Temperature scale) of the research site on the Island of Aegina, from January (J) to December (D); the bars represent the mean monthly values of the precipitation and the black line the mean monthly values of the temperatures, during the year 2021.

The first year of the experimental research (i.e., 2021) was conducted under controlled conditions (CCs); *Vitis vinifera* cv. Assyrtiko plants, newly emerged shoots with 4–5 buds, were grown from canes approximately 50 cm tall in pots (6 L) containing a mixture of 55% sand, 30% clay, 15% silt, and 1.5% organic matter. The pH of the substrate ranged from 7.5 to 8.0. Eighty potted grapevines *Vitis vinifera* cv. Assyrtiko grafted on 1103 Paulsen (*V*. *rupestris* × *V*. *berlandieri*, rootstock 1103P) were initially transferred to a growth chamber (CCs: controlled conditions), in the Biology Department at the National and Kapodistrian University of Athens (Greece), and were subjected to controlled conditions, i.e., 14 °C, with a 15 h photoperiod (500 µmol m^2^ s^−1^ PAR), and 60% relative humidity for two weeks [6]. Then, the plants were divided in four groups (each group was consisted of 20 plants), i.e., control plants (Cs) subjected to controlled conditions (C-CCs), plants subjected to water deficit (WD-CCs), plants exposed to elevated temperature (ET-CCs), and plants exposed to a combination of water deficit and elevated temperature conditions (WD_ET-CCs). The selected experimental conditions, i.e., elevated temperature and reduced irrigation, correspond to the last decade’s mean monthly values of temperature and precipitation obtained from the Island of Aegina (Figure 8), where the experimental research of the second year was performed, between April and September (i.e., the leafy period of grapevines); climatic data from a nearby station provided by the Hellenic National meteorological services (37°44′53.3″ N, 23°26′37.1″ E) have been used (Figure 8 and Figure 9). During the period from April to September the mean air temperature and the precipitation were 25 ± 0.3 °C and 7 ± 2 mm, respectively.

The C-CC plants were watered to run-off and allowed to drain to soil capacity. The WD-CC plants were exposed to an irrigation 30% lower than that of the control plants (C-CCs); the selected reduction in irrigation by 30% is consistent with recent projections for precipitation over Greece [84]. The ET-CC plants were placed into a growth chamber with an elevated temperature (14 + 2 °C), consistent with IPCC projections [85,86], and were adequately watered, ensuring no runoff. The WD_ET-CC plants were also placed in the chamber with an elevated temperature and were subjected to the reduced (by 30%) irrigation, as were the WD-CC plants. The photoperiod, light intensity, and humidity in the growth chambers remained constant (as mentioned above) for four months.

#### 4.1.2. Greenhouse and Field Conditions

In the second year (2022), the experiments were conducted in an open-air vineyard, and an adjacent greenhouse on Aegina Island (Greece), where eighty *Vitis vinifera* cv. Assyrtiko two-year-old plants (rootstock 1103P) were transplanted and maintained growing in a greenhouse (GCs: greenhouse conditions) and a neighboring vineyard (FCs: field conditions) located in Agia Marina on Aegina Island in Greece (37°44′51.7″ N, 23°32′19.0″ E), for twelve months prior to the imposed experimental procedures. The soil was a mixture of 63.2% sand, 19.4% clay, and 17.4% silt. The average PAR was approximately 500 μmol m^−2^ s^−1^ [87]. The distances between the planting rows were 2.2 m and 1.2 m among grapevines of the same row, according to the unilateral Guyot system [88], in the vineyard that was established in January 2022. Monthly mean temperature and monthly precipitation at the study site were obtained from the closest meteorological station provided by the Hellenic National Meteorological Service. Inside the greenhouse, the air temperature was 2–3 °C higher than that of the ambient, open-air conditions in the vineyard. Prior to the measurements, the predawn relative humidity (RH) in the greenhouse varied from 70% to 80% [89]. Also, the mean annual RH in the surrounding, ambient environment of the research site was estimated at 65–75% [22]. The windows and the door of the greenhouse were manually opened at sunrise and closed at sunset; thus, the RH in the greenhouse declined during daytime.

Eighty grapevine plants were divided in four groups and exposed to different environmental conditions, mirroring the growth chamber conditions of the first year experimental research; i.e., control plants (Cs), plants subjected to water deficit (WD), to elevated temperature (ET), and to the combined effect of water deficit and elevated temperature (WD_ET). The control plants (C-FCs) exposed to ambient temperature in the vineyard were adequately watered (well-watered), using low-volume drip irrigation without causing runoff [90], where soil moisture was generally saturated. The plants subjected to water deficit (WD) were grown under ambient temperature (WD-FC), and exposed to a reduction in irrigation by 30% compared to that of the control plants, according to grapevine evapotranspiration (ETc), which is the potential amount of water lost to the atmosphere from the given cultivar Assyrtiko and its surrounding soil under prevailing agro-meteorological conditions [42].

The well-watered (C-FC) plants were supplied with 10 L of tap water, while the plants subjected to water deficit (WD-FC) were irrigated with 7 L of tap water. The plants subjected to elevated temperature (ET) were grown in the neighboring greenhouse (ET-GC), where the temperature was higher (+2 °C) than that of the ambient conditions according to relevant projection at 2 °C additional warming level [16,84,85,91,92,93] and were irrigated as the control plants. The plants exposed to a combination of water deficit and elevated temperature (WD_ET) were also placed in the greenhouse (WD_ET-GC) with the higher temperature (+2 °C) and were subjected to irrigation reduced by 30%.

### 4.2. Leaf Material

Young expanding leaves were collected from the second and/or the third node of the stem, while fully expanded leaves were collected from the eight and/or the ninth node [94]; leaf sampling of control plants (Cs), as well as of plants subjected to WD, ET, and WD_ET treatments and exposed to CCs, GCs, and FCs was performed simultaneously. Young expanding and fully expanded leaves were selected from control and treated plants in the morning (from 7 to 8 a.m.) for physiological, biochemical, and anatomical observations. The cut petioles were wrapped with water-soaked cotton and aluminum foil. After sampling, the material was rapidly transported to the Department of Biology (National and Kapodistrian University). In the laboratory, the leaves were scanned in a flatbed scanner to calculate their fresh area ImageJ Pro Plus (v.5.1.0.20) and then oven-dried at 60 °C for 48 h to a constant mass and weighed to the nearest 0.001 g. Then, the dry plant material was pulverized using a grinding mill (Janke & Kunkel-Mikro-Feinmuhle Cullati, IKA Labortechnik, Schönwalde-Glien, Germany) and stored in airtight storage containers, in the dark.

### 4.3. Leaf Water Potential

The predawn water potential (Ψ_leaf_) of fully expanded leaves collected from C, WD, ET, and WD_ET plants grown under GCs and FCs (Table 6) was measured using a Skye Scholander-type pressure chamber (Skye Instruments Ltd., Llandrindod Wells, UK). The leaves were placed in the pressure chamber, where the pressure was gradually increased at a constant rate (~0.01 MPa s^−1^). The pressure at which a water meniscus first appeared on the cut petiole surface was recorded as the value of the leaf water potential; each value is the mean of seven measurements from seven leaves from different plants.

It is interesting to note that predawn Ψ_leaf_ is often used as a proxy for soil water potential (which is difficult to quantify due to heterogeneity of the water distribution in the soil [95] and water flux into the roots was assumed to be either comparable or equal to the water potential of the leaves [96]. Also, the values of predawn Ψ_leaf_ (Table 5) in the case of the experimental site on Aegina Island indicate underlying assumptions, i.e., that during the night a quite similar leaf hydraulic equilibrium was achieved according to the root density distribution (of the same age grapevines) and the available soil water content in a relatively similar wet range and soil depth, between the greenhouse and the neighboring vineyard.

### 4.4. Microscopy

Samples from the central area of the leaf blade fragments were chemically stabilized using a modified Karnovsky’s fixative [97], in a solution containing 2% *w*/*v* paraformaldehyde (PFA) and 3% *v*/*v* glutaraldehyde (GA), in 0.05 M sodium cacodylate buffer (pH 7.0) for 3 h with a constant mixing procedure, at room temperature. After fixation, the samples were rinsed three times, each for 15 min, in the same buffer. The tissues were then dehydrated through a graded ethanol series and post-fixed in 1% OsO_4_ in the same buffer, followed by further dehydration using propylene oxide. The dehydrated samples were embedded in LR white resin and cured for 72 h at 60 °C. The embedded samples were sectioned using an ultramicrotome (ULTROTOME III Type 8801A, LKB, Stockholm, Sweden) fitted with a glass knife. Sections were mounted on glass slides using Entellan mounting medium (Sigma-Aldrich, Burlington, MA, USA). Semi-thin sections (0.5–2.0 μm) were stained with 0.5% *w*/*v* toluidine blue “O”, used for nuclei and different components of the cell wall (general polychromatic staining) [98], and observed using a Zeiss Axioplan light microscope (Carl Zeiss AG, Göttingen, Germany) equipped with a digital AxioCam MRc 5 camera [99]. Photographs were taken using the digital camera (magnification: 20 μm).

Leaf traits, including leaf thickness (μm), spongy mesophyll thickness (μm), and palisade mesophyll thickness (μm), were measured using the ImageJ (1.51d) software. Concerning the intercellular area (μm^2^), intercellular regions were selected by manual tracing, and the areas were measured with the ImageJ software (https://imagej.net/ij/, accessed on 5 July 2025) [100] using the “Analyze–Measure” menu. Quantitative measurements were taken from three different samples; ten photographs taken from three different samples for each treatment and developmental stage were analyzed. In each photograph ten measurements of each parameter were calculated.

### 4.5. Specific Leaf Area

Specific leaf area (SLA) was calculated as the ratio of the fresh leaf area versus the dry mass (cm^2^ g^−1^). Ten young expanding and ten fully expanded leaves from plants of each treatment (i.e., C, WD, ET, and WD_ET) grown under CCs, GCs, and FCs were collected for the determination of SLA. The leaves were immediately scanned in a flatbed scanner to calculate the fresh leaf area using the ImageJ (1.51d) software; then, the leaves were oven-dried at 60 °C for 48 h to a constant mass and weighed to the nearest 0.001 g. The results are means of measurements from ten samples ± standard error (S.E.) [99].

### 4.6. Soluble Sugars and Starch

Soluble sugars were extracted from dry, finely powdered leaf samples, which were placed in 10 mL 80% ethanol (*v*/*v*), in a shaker, and the extracts were filtered using Whatman # 2 filter paper. The soluble sugar concentration was investigated according to a modified colorimetric method [67,101], at 490 nm, using a spectrophotometer (Novaspec III+, Biochrom, Cambridge, UK). The determination of starch was made in the residue after the extraction of sugars, using a colorimetric method [67,102] and was investigated colorimetrically at 600 nm using the Novaspec spectrophotometer. D-glucose (Serva, Heidelberg, Germany) aqueous solutions (0, 25, 50, and 100 μg mL^−1^) were used for the standard curve. The analyses were performed in triplicate. The values are expressed as mg g^−1^ d.w. The results are means of five replicates ± standard error.

### 4.7. Phenolic Content

The phenolic content in leaf extracts was determined using the Folin–Ciocalteu colorimetric method [103,104,105,106]. Briefly, 0.1 g of dry pulverized leaf tissues was immersed in 10 mL MeOH (50% *v*/*v*), incubated in a water bath for 3 h at 40 °C, and vortexed regularly. Phenolic compounds were extracted, filtered (Whatman #2 filter paper, Sigma-Aldrich Chemie GmbH, Munich, Germany), and kept tightly sealed at 4 °C overnight. An aliquot (0.05 mL) of the diluted leaf extract [1:5 MeOH 10% (*v*/*v*)] was added to 3.95 mL of dH_2_O, 0.25 mL Folin–Ciocalteu reagent (Sigma-Aldrich Chemie GmbH, Munich, Germany), which was previously diluted with water 1:10 *v*/*v*, 0.75 mL Na_2_CO_3_ 20% (*w*/*v*), and vortexed. The solution was maintained at 20 °C for 2 h and the absorption of the resulting colorimetric reaction was measured with a UV–VIS spectrophotometer (Novaspec III+, Biochrom, Cambridge, UK) at 760 nm. The total phenolic content was calculated using the standard curve of tannic acid and expressed as mg of tannic acid equivalent per g of leaf dry weight. For the calibration curve, 0.05 mL of a gradient of tannic acid concentrations (0.02 mg mL^−1^, 0.08 mg mL^−1^, 0.16 mg mL^−1^, and 0.40 mg mL^−1^) were added to 3.95 mL of dH_2_O, 0.25 mL Folin–Ciocalteu reagent (previously diluted with distilled water 1:10 *v*/*v*), 0.75 mL Na_2_CO_3_ 20% (*w*/*v*), and vortexed. The absorbance of the resulting fluids of the colorimetric reaction (after 2 h at 20 °C) was measured with the above-mentioned UV–VIS spectrophotometer at 760 nm. The phenolic content quantification was achieved by plotting the recorded absorbance versus the relevant concentration of the standard solutions. The results are means of five replicates ± standard error.

### 4.8. Leaf Plasticity Index

The leaf plasticity index (LPI) was calculated for each measured variable as the difference between maximum and minimum values divided by the maximum value [107]. The LPI ranges from 0 value (no plasticity) to 1 indicating high plasticity.

### 4.9. Statistical Analyses

The statistical analysis was conducted using the Python programming language (version 3.9.16). To assess the normality and homoscedasticity of the data, the Shapiro–Wilk test and Levene’s test were performed, respectively. These tests were applied to ensure that the assumptions for subsequent analyses were met. Based on the results of the tests, data transformation was not required. A two-way ANOVA was conducted in order to examine the effect of experimental treatments on leaf biochemical, morphological, and anatomical features for young expanding and fully expanded leaves. Post hoc comparisons between means were performed using Tukey’s HSD (Honestly Significant Difference) test in order to identify significant differences between treatment groups. Furthermore, a mixed-effects model analysis was performed to evaluate the effects of drought, elevated temperature, and their interactions in order to analyze the potential variability in the leaf features according to the leaf developmental status. This analysis was used to assess the influence of fixed effects (e.g., leaf expansion, treatment) while accounting for random effects (e.g., time). The results of the mixed-effects model further supported the significance of the observed treatments’ effects. The significance level of the statistical procedures was set at 0.05. Principal component analysis (PCA) was used to visualize the differences and similarities between leaf developmental stages, as well as to confirm analytical variability within the data. PCA was performed on biochemical, morphological, and anatomical data using the software OriginPro10 from Origin Labs. The data were centered to normalize the variables and ensure that the considered qualities were at the same scale.

## 5. Conclusions

Under ET conditions, the SLA of young expanding leaves increased, while the SLA of fully expanded leaves increased under WD, ET, and WD_ET. Young leaves maintained a similar thickness under the studied stress conditions, whereas fully expanded leaves became thicker under WD and ET. It is likely that these changes reflect the adaptive tactics of *Vitis vinifera* cv. Assyrtiko leaves. Soluble sugar content enhanced in young expanding leaves under the studied stress conditions, aiding in osmotic adjustment and their turgor maintenance. Meanwhile, starch content decreased in young expanding and fully expanded leaves, indicating a mobilization of carbohydrate reserves. The reduced phenolic content suggests either a limitation or a tolerance in countering oxidative stress. Fully expanded leaves showed more stable traits than young expanding leaves, highlighting the importance of leaf development of *Vitis vinifera* cv. Assyrtiko to climate change. Grape production is based on leaf function in order to keep vines and wines under control; also, the search to augment grape quantity and quality is frequently linked to the desire of vine producers and consumers for traditional and tolerant cultivars, such as the Assyrtiko grape of Santorini.

## Figures and Tables

**Table 1:** Thickness of leaf, palisade, and spongy parenchyma, and intercellular areas of young expanding leaves of *Vitis vinifera* cv. Assyrtiko. Quantitative measurements were taken from three different samples (n = 3); the values are means ± standard error. Significant differences (*p* < 0.05) of mean values are marked using lowercase superscript letters that are given separately on each column variable.

	Young Expanding Leaves			
	Controlled Conditions (CCs)			
	Area	Thickness	Thickness	Thickness
Treatment	Intercellular (μm^2^)	Palisade Parenchyma (μm)	Spongy Parenchyma (μm)	Leaf (μm)
Control	298.02 ^a^ ± 171.11	18.67 ^a^ ± 0.73	40.50 ^a^ ± 4.95	76.92 ^a^ ± 4.52
WD	173.05 ^b^ ± 59.86	17.84 ^a^ ± 2.35	39.25 ^a^ ± 1.93	77.54 ^a^ ± 2.30
ET	54.93 ^c^ ± 26.15	16.37 ^bc^ ± 1.40	35.37 ^b^ ± 1.66	74.60 ^a^ ± 3.15
WD_ET	83.94 ^bc^ ± 23.23	16.37 ^bc^ ± 1.22	34.88 ^b^ ± 2.59	72.59 ^a^ ± 1.88
	**Field Conditions (FCs)–Greenhouse Conditions (GCs)**			
Control	169.11 ^a^ ± 30.54	20.09 ^a^ ± 3.53	40.28 ^a^ ± 6.49	76.09 ^a^ ± 8.02
WD	172.90 ^a^ ± 83.33	20.02 ^a^ ± 2.62	37.33 ^a^ ± 4.74	72.38 ^a^ ± 8.31
ET	57.04 ^c^ ± 22.94	21.79 ^a^ ± 0.88	35.54 ^a^ ± 1.96	70.80 ^a^ ± 2.39
WD_ET	72.55 ^b^ ± 23.56	20.93 ^a^ ± 2.96	37.52 ^a^ ± 6.78	75.78 ^a^ ± 9.71

**Table 2:** Thickness of leaf, palisade, and spongy parenchyma, and intercellular area of fully expanded leaves of *Vitis vinifera* cv. Assyrtiko. Quantitative measurements were taken from three different samples (n = 3); the values are means ± standard error. Significant differences (*p* < 0.05) of mean values are marked using lowercase superscript letters that are given separately on each column variable.

	Fully Expanded Leaves			
	Controlled Conditions (CCs)			
	Area	Thickness	Thickness	Thickness
Treatment	Intercellular (μm^2^)	Palisade Parenchyma (μm)	Spongy Parenchyma (μm)	Leaf (μm)
Control	256.67 ^cb^ ± 101.12	18.84 ^a^ ± 1.65	42.17 ^ab^ ± 5.65	80.97 ^bc^ ± 5.76
WD	416.81 ^ac^ ± 82.44	18.27 ^a^ ± 1.31	47.88 ^b^ ± 6.80	87.84 ^b^ ± 6.53
ET	443.74 ^a^ ± 72.60	17.21 ^a^ ± 2.50	56.55 ^a^ ± 4.90	96.48 ^a^ ± 6.54
WD_ET	183.53 ^bcd^ ± 66.12	14.10 ^bc^ ± 4.34	35.35 ^c^ ± 9.80	70.69 ^cd^ ± 4.58
	**Field Conditions (FCs)–Greenhouse Conditions (GCs)**			
Control	258.71 ^a^ ± 93.62	29.61 ^a^ ± 2.07	39.47 ^c^ ± 2.65	86.92 ^bc^ ± 2.20
WD	283.40 ^a^ ± 92.83	30.40 ^a^ ± 2.58	47.62 ^b^ ± 5.94	92.44 ^b^ ± 10.05
ET	201.76 ^ab^ ± 90.89	26.62 ^a^ ± 3.82	62.81 ^a^ ± 2.50	101.92 ^a^ ± 4.17
WD_ET	126.24 ^b^ ± 56.99	22.80 ^a^ ± 2.58	39.14 ^c^ ± 2.90	79.22 ^bc^ ± 3.72

**Table 3:** Plasticity indices (PIs), given in a 0–1 scale, for morphological and metabolic traits of young expanding and fully expanded leaves of *Vitis vinifera* cv. Assyrtiko control plants (Cs) and plants exposed to water deficit (WD), elevated temperature (ET), and a combination of water deficit and elevated temperature (WD_ET), and subjected to CCs (controlled conditions) (A) and FC-GCs (field conditions and greenhouse conditions).

Young Expanding Leaves Under Controlled Conditions (CCs)								
	Area Intercellular (μm^2^)	Thickness Palisade Parenchyma (μm)	Thickness Spongy Parenchyma (μm)	Thickness Leaf (μm)	Phenols (mg g^−1^)	Sugar (mg g^−1^)	Starch (mg g^−1^)	SLA (cm^2^ g^−1^)
C	0.78	0.11	0.31	0.17	0.44	0.81	0.74	0.22
WD	0.75	0.34	0.15	0.16	0.52	0.93	0.58	0.36
ET	0.76	0.24	0.15	0.14	0.53	0.74	0.65	0.54
WD_ET	0.69	0.24	0.21	0.14	0.48	0.71	0.64	0.38
**Young expanding leaves under** **Field Conditions (FC)-Greenhouse Conditions (GC)**								
C	0.95	0.38	0.37	0.26	0.59	0.37	0.66	0.38
WD	0.75	0.30	0.31	0.27	0.68	0.31	0.24	0.76
ET	0.79	0.21	0.16	0.20	0.69	0.33	0.49	0.77
WD_ET	0.89	0.28	0.44	0.32	0.56	0.53	0.53	0.81
**Fully expanded leaves under Controlled Conditions (CC)**								
C	0.70	0.24	0.33	0.19	0.22	0.67	0.71	0.34
WD	0.76	0.21	0.37	0.20	0.20	0.62	0.64	0.21
ET	0.83	0.39	0.24	0.22	0.33	0.63	0.78	0.61
WD_ET	0.72	0.60	0.62	0.64	0.24	0.72	0.68	0.51
**Fully expanded leaves under** **Field Conditions (FCs)–Greenhouse Conditions (GCs)**								
C	0.72	0.22	0.23	0.07	0.46	0.21	0.53	0.57
WD	0.85	0.28	0.28	0.23	0.25	0.25	0.66	0.73
ET	0.80	0.35	0.27	0.21	0.25	0.26	0.61	0.71
WD_ET	0.73	0.32	0.21	0.16	0.32	0.14	0.71	0.36

**Table 4:** Mixed-effects model analysis of leaf structural and biochemical parameters in response to leaf developmental stage and treatment. The model included leaf expansion as a fixed effect and time as a random effect. Intercept coefficients (C_oefs_) represent baseline values, while leaf DS coefficients indicate the change in parameters for young expanding leaves relative to fully expanded leaves. Standard errors (S.E.) and *p*-values are provided for both intercept and age effects. Group variance represents the random variance effect attributed to treatments.

Variable	Intercept C_oef_	Intercept *p*-Value	Leaf DS C_oef_	Leaf DS S.E.	Leaf DS *p*-Value	Group Variance (Treatment)
Phenols	0.394	0.001	0.011	0.034	0.742	0.000
Sugars	74.714	0.001	−9.827	7.624	0.198	0.062
Starch	62.175	0.001	−11.175	1.927	0.001	62.532
Intercellular Space	271.190	0.001	−139.589	18.334	0.001	3416.514
Palisade Parenchyma	22.213	0.001	−3.223	0.672	0.001	1.786
Spongy Parenchyma	43.022	0.001	−5.438	0.942	0.001	5.984
Leaf Thickness	83.814	0.001	−9.226	1.323	0.001	9.507
SLA	101.351	0.001	15.284	12.028	0.204	596.286

**Table 5:** Effect of water deficit (WD), elevated temperature (ET), and combination of water deficit and elevated temperature (WD_ET) on leaf developmental stage (DS) for different dependent variables. Mixed-effects model was used with leaf DS as fixed effect.

Dependent Variable	Treatment WD Leaf DS (*p*-Value)	Treatment ET Leaf DS (*p*-Value)	Treatment WD_ET Leaf DS (*p*-Value)
Leaf Thickness	0.031	0.090	0.063
Palisade Parenchyma	0.668	0.097	0.001
Spongy Parenchyma	0.001	0.001	0.803
Intercellular Space	0.024	0.001	0.874
SLA	0.003	0.076	0.001
Phenols	0.113	0.082	0.024
Sugars	0.055	0.880	0.225
Starch	0.532	0.255	0.569

**Table 6:** Leaf water potential (Ψ_leaf_) of fully expanded leaves of *Vitis vinifera* L. cv. Assyrtiko, control (C) and treated (WD, ET, and WD_ET) plants grown under greenhouse (GC) and field (FC) conditions. The values are means of seven replicates (n = 7) ± standard error. Significant differences (*p* < 0.05) of mean values are presented using exponent, lowercase letters.

Leaf Water Potential (Ψ_leaf_, MPa)		
Treatments	GC	FC
C (control)	−1.25 ^b^ ± 0.007	−1.24 ^b^ ± 0.010
WD (water deficit)	−1.58 ^c^ ± 0.003	−1.60 ^d^ ± 0.005
ET (elevated temperature)	−0.95 ^a^ ± 0.001	−0.97 ^a^ ± 0.011
WD_ET (water deficit and elevated temperature)	−1.53 ^c^ ± 0.009	−1.49 ^c^ ± 0.007

## Data Availability

Data are contained within the article.
